# A Rare Case of a Primary Isolated Intraperitoneal Teratoma With a Yolk Sac Tumor Component in a Three-Year-Old Boy

**DOI:** 10.7759/cureus.95115

**Published:** 2025-10-21

**Authors:** Raj M Dongol, Neera Pathak, Moni Subedi

**Affiliations:** 1 Department of Pathology and Laboratory Medicine, George Washington University School of Medicine and Health Sciences, Washington, DC, USA; 2 Department of Pathology, Kanti Children Hospital, Kathmandu, NPL

**Keywords:** intraperitoneal, isolated, primary, teratoma, yolk sac tumor

## Abstract

Primary isolated intraperitoneal teratoma is an exceptionally rare entity, and the presence of a yolk sac tumor component within it is even more uncommon. We report a case of a three-year-old boy diagnosed with a primary isolated intraperitoneal teratoma containing a yolk sac tumor component. The patient underwent complete surgical excision followed by adjuvant chemotherapy, with a favorable clinical outcome. Histopathological evaluation of teratomas with malignant components poses a significant diagnostic challenge, underscoring the critical role of identifying malignant elements such as yolk sac tumor to guide appropriate management.

## Introduction

Germ cell tumors (GCTs) arise due to aberrant migration of germ cells from the yolk sac during fetal development and can therefore occur anywhere in the body. These are rare tumors, with an incidence of about 12 cases per one million individuals under the age of twenty [1]. GCTs can be subdivided into teratoma, seminoma (known as dysgerminoma in the ovary and germinoma in the pineal gland), choriocarcinoma, yolk sac tumor, embryonal carcinoma, and mixed GCT [2]. In the pediatric population, teratomas are the most common type of germ cell tumor [3]. Teratomas contain tissues derived from two or more germ layers-ectoderm, mesoderm, and endoderm. They can be classified as mature, immature, or mixed types based on their histological components. In adults, teratomas are most commonly found in the gonads, whereas in children, sacrococcygeal teratomas predominate, followed by gonadal tumors [2,4]. Other sites, in decreasing order of frequency, include the anterior mediastinum, retroperitoneum, cranium, and intraperitoneal locations [4].

Extragonadal abdominopelvic teratomas are rare tumors, representing only about 1-5% of all germ cell tumors [1]. Primary isolated extragonadal intraperitoneal teratoma with a malignant yolk sac tumor component is an extremely rare entity. We present the case of a three-year-old boy diagnosed with this unusual tumor, managed successfully with complete surgical excision followed by adjuvant chemotherapy. This case underscores the importance of including germ cell tumors in the differential diagnosis of isolated intraperitoneal masses without any apparent visceral origin. Early diagnosis through appropriate evaluation and timely management is crucial, as the malignant component of such tumors has the potential to metastasize. Prompt surgical removal combined with postoperative chemotherapy can greatly improve survival outcomes in these patients.

## Case presentation

**Figure 1:**
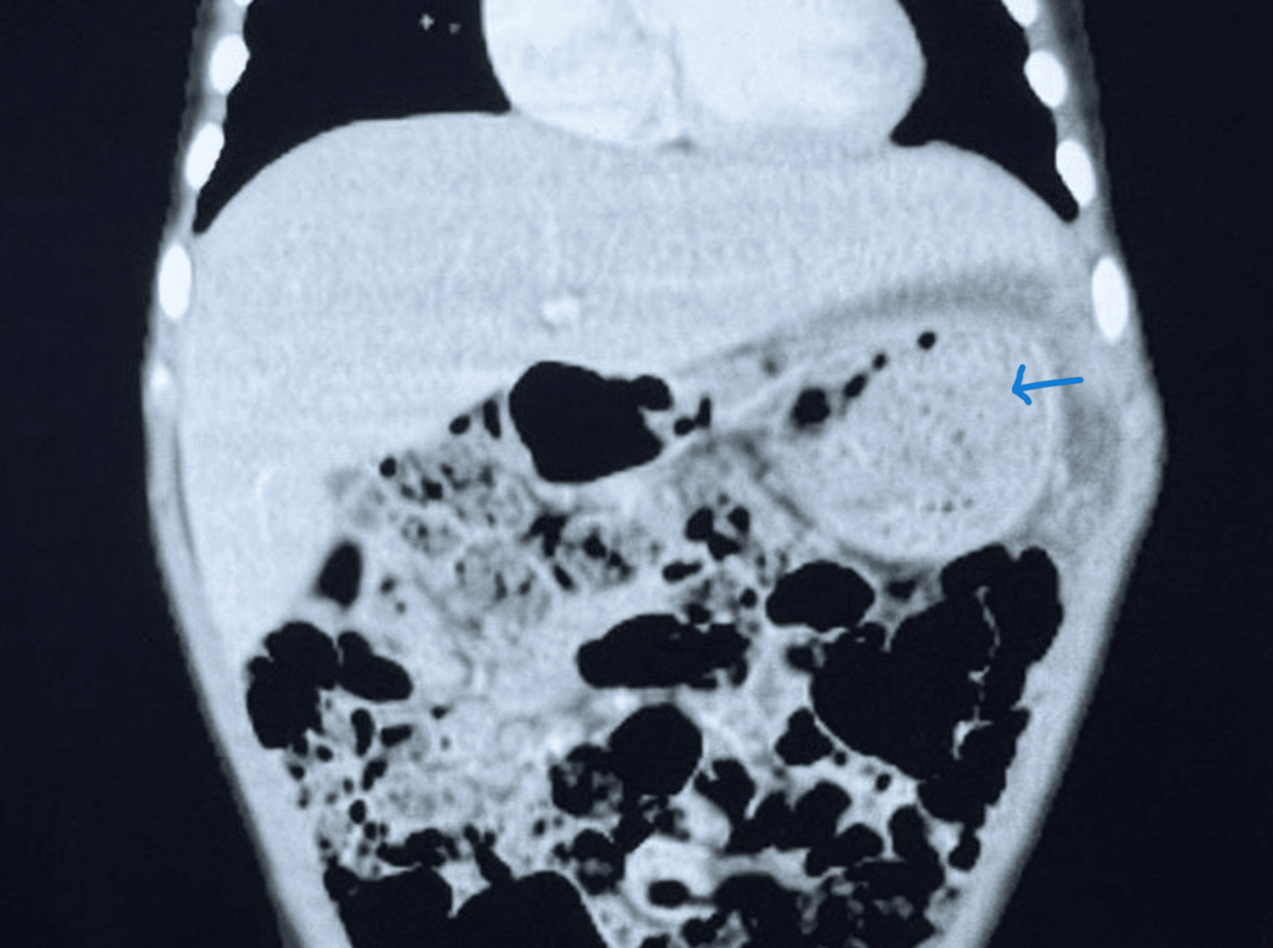
Coronal contrast-enhanced computed tomography (CT) of the abdomen Computed tomography (CT) of abdomen showing a 13 × 12 × 7.6 cm well-defined multiloculated cystic mass (blue arrow) with enhancing solid nodule and septa, superiorly abutting the diaphragm, laterally abutting the abdominal wall, and medially displacing the bowel loops, pancreas, and stomach.

**Figure 2:**
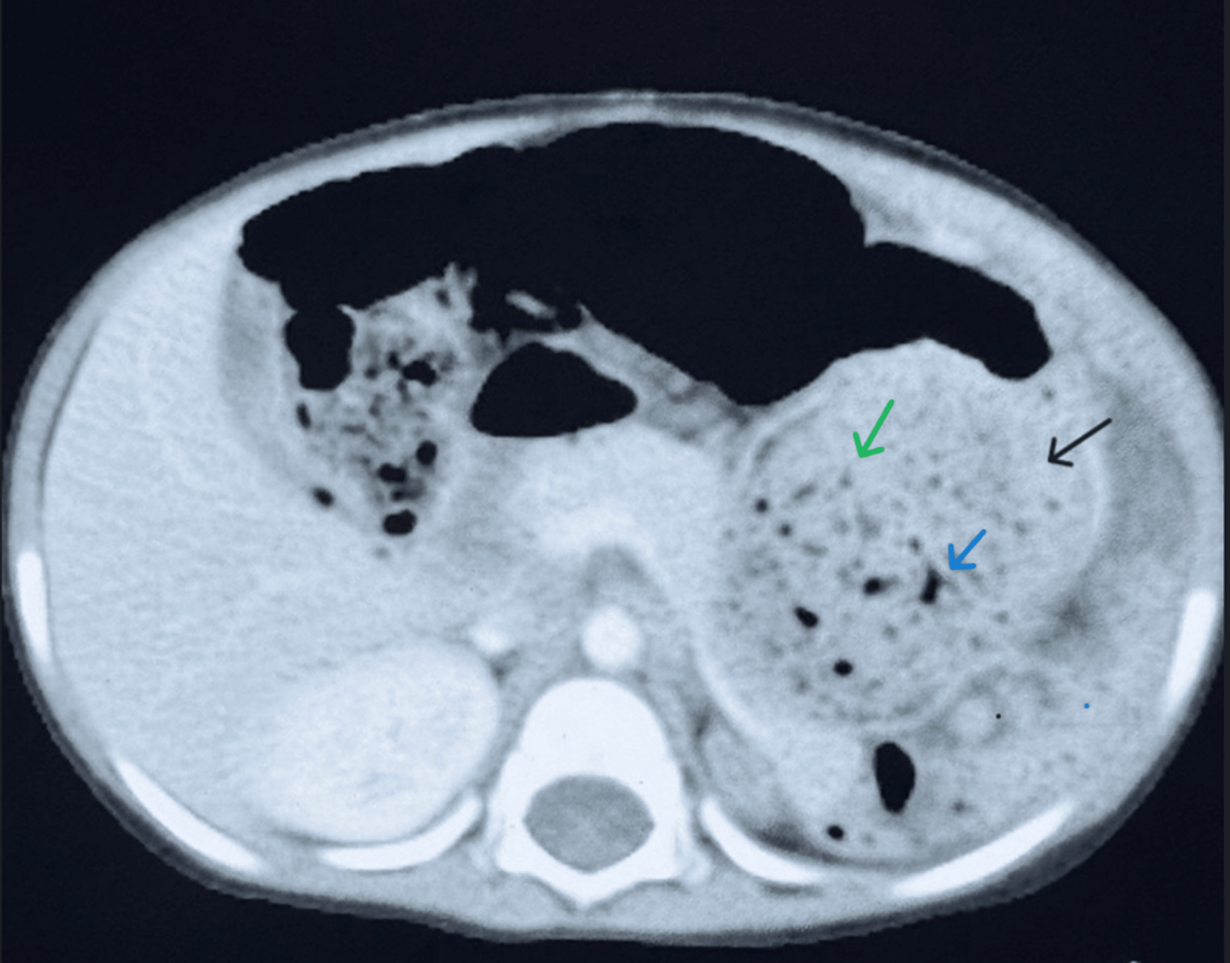
Axial contrast-enhanced computer tomography (CECT) of the abdomen Axial CT image showing a well-defined multiloculated cystic mass (black arrow) with enhancing septa (blue arrow) and solid component (green arrow) in the left upper abdomen, laterally and posteriorly abutting the spleen, anterolaterally abutting the abdominal wall, and medially displacing adjacent bowel loops, pancreas and stomach.

A three-year-old boy presented with a left upper quadrant abdominal mass that was first noticed at the age of two. The swelling had gradually increased in size over the course of one year, but was initially ignored by the family. On examination, the general physical findings were unremarkable, and the patient’s vital signs were stable. Abdominal examination revealed a lump in the left upper quadrant measuring approximately 15 × 10 cm. The mass moved with respiration, was non-mobile, had a smooth surface and well-defined margins, and was non-tender. Examination of other systems was unremarkable. Computed tomography (CT) of the abdomen revealed a 13 × 12 × 7.6 cm well-defined multiloculated cystic mass with an enhancing solid nodule and septa encasing mesenteric fat (Figures 1, 2). Superiorly, it was abutting the diaphragm; posteriorly, the spleen; and anterolaterally, the abdominal wall. The bowel loops, pancreas, and stomach were displaced medially by the mass. A differential diagnosis of teratoma versus lymphangiosarcoma was considered. Ultrasonography of the bilateral testes was normal. Preoperative serum tumor markers showed elevated alpha-fetoprotein (AFP, 13258 IU/mL) and beta-human chorionic gonadotropin (β-hCG, 0.3 mIU/L). An exploratory laparotomy with complete excision of the mass (without removal of any other visceral organ or part thereof) was performed, and the specimen was sent for histopathological examination (HPE). Intraoperatively, a 17 × 15 × 12 cm solid-cystic mass (isolated, without origin from any visceral structure) was found in the left upper quadrant, indenting the diaphragm superiorly, adherent to the greater curvature of the stomach medially, abutting the spleen laterally, and compressing the kidney inferiorly.

**Figure 3:**
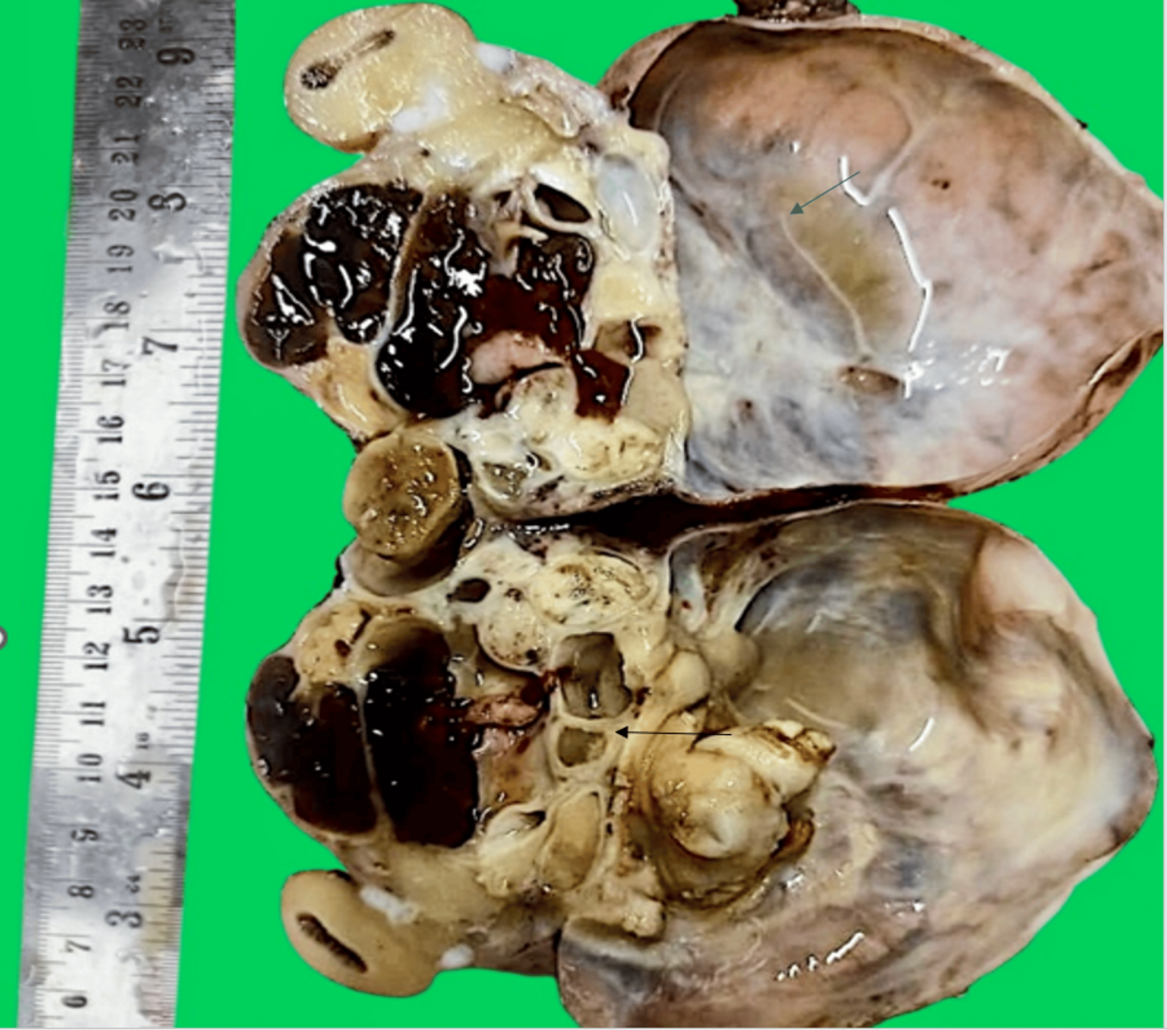
Cut surface of tumor The image is showing a cystic (blue arrow) and a solid area (black arrow).

Grossly, the tumor was globular, well-encapsulated, and gray-brown to blackish in color with multiple bosselations. The cut surface showed a solid-cystic appearance, with cysts ranging in size from 1.2 to 10 cm, filled with brownish to yellowish material. The cyst walls were 0.1 to 0.3 cm thick. The solid area measured up to 9 × 2.5 cm. Representative sections were taken. Gross features are shown in Figure 3.

**Figure 4:**
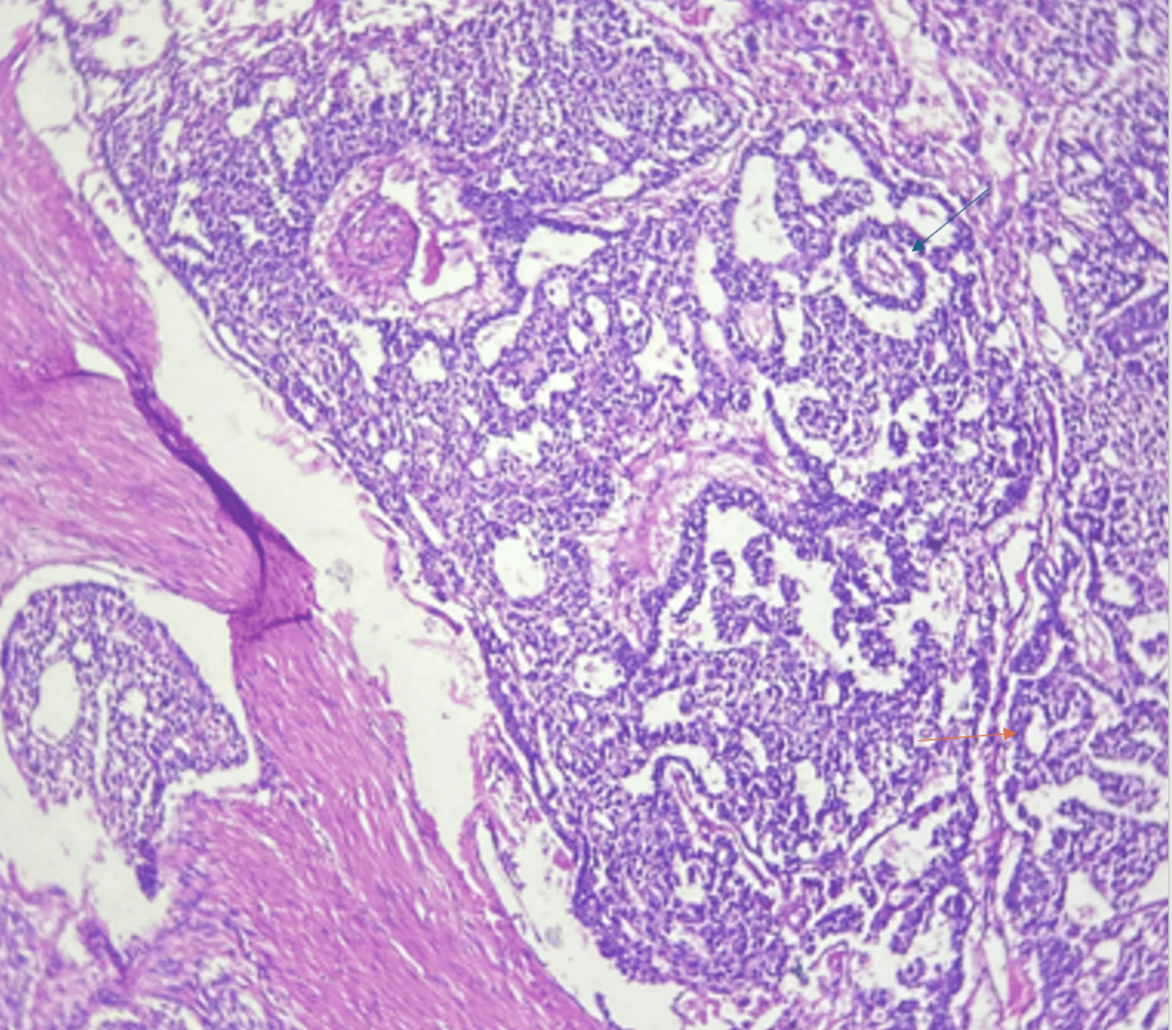
Low power view (10X magnification) of yolk sac tumor The image is showing Schiller-Duval body (blue arrow) and microscystic pattern (orange arrow).

**Figure 5:**
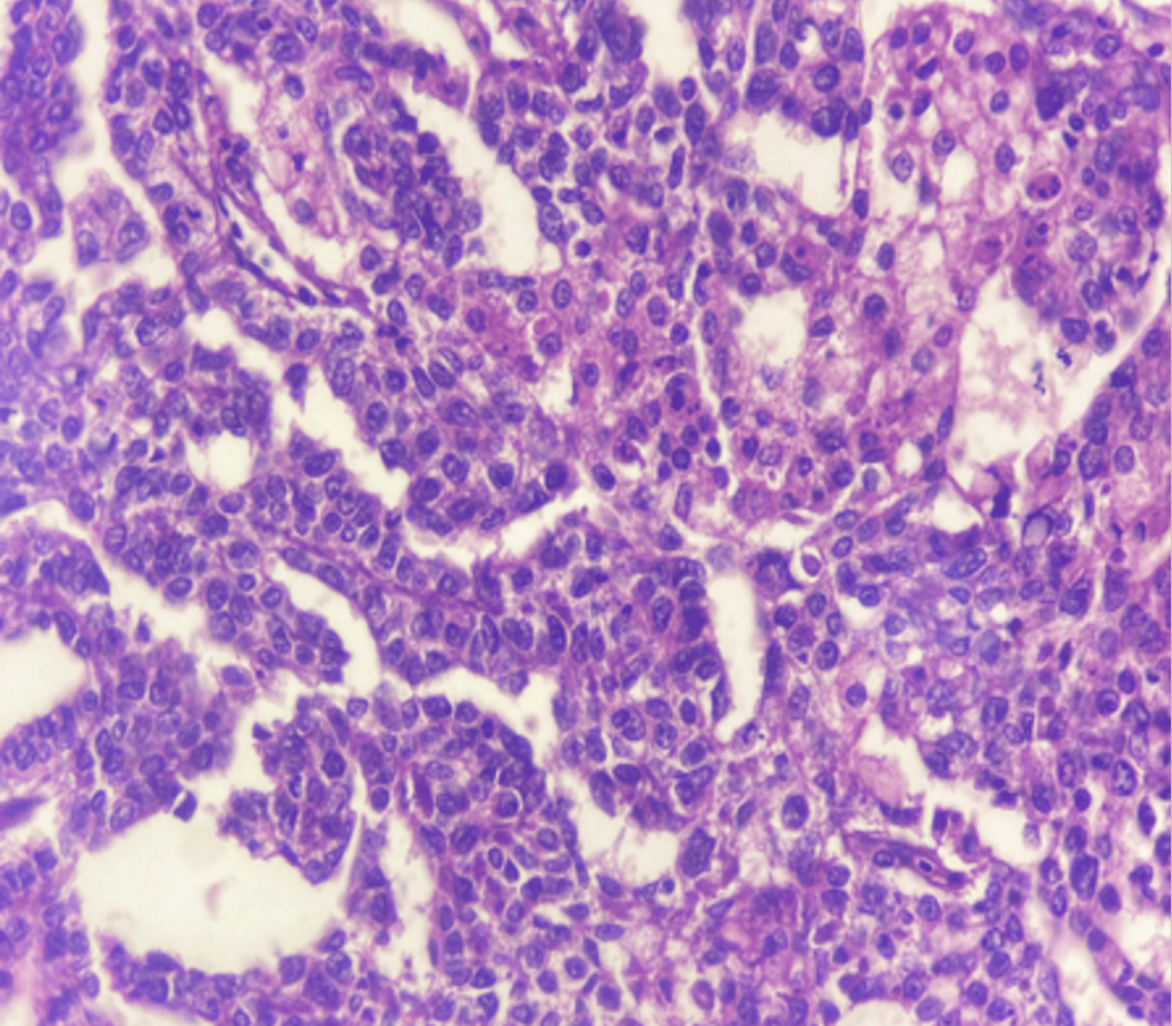
High power view (40X magnification) of yolk sac tumor The image is showing atypical cells forming microcystic pattern.

**Figure 6:**
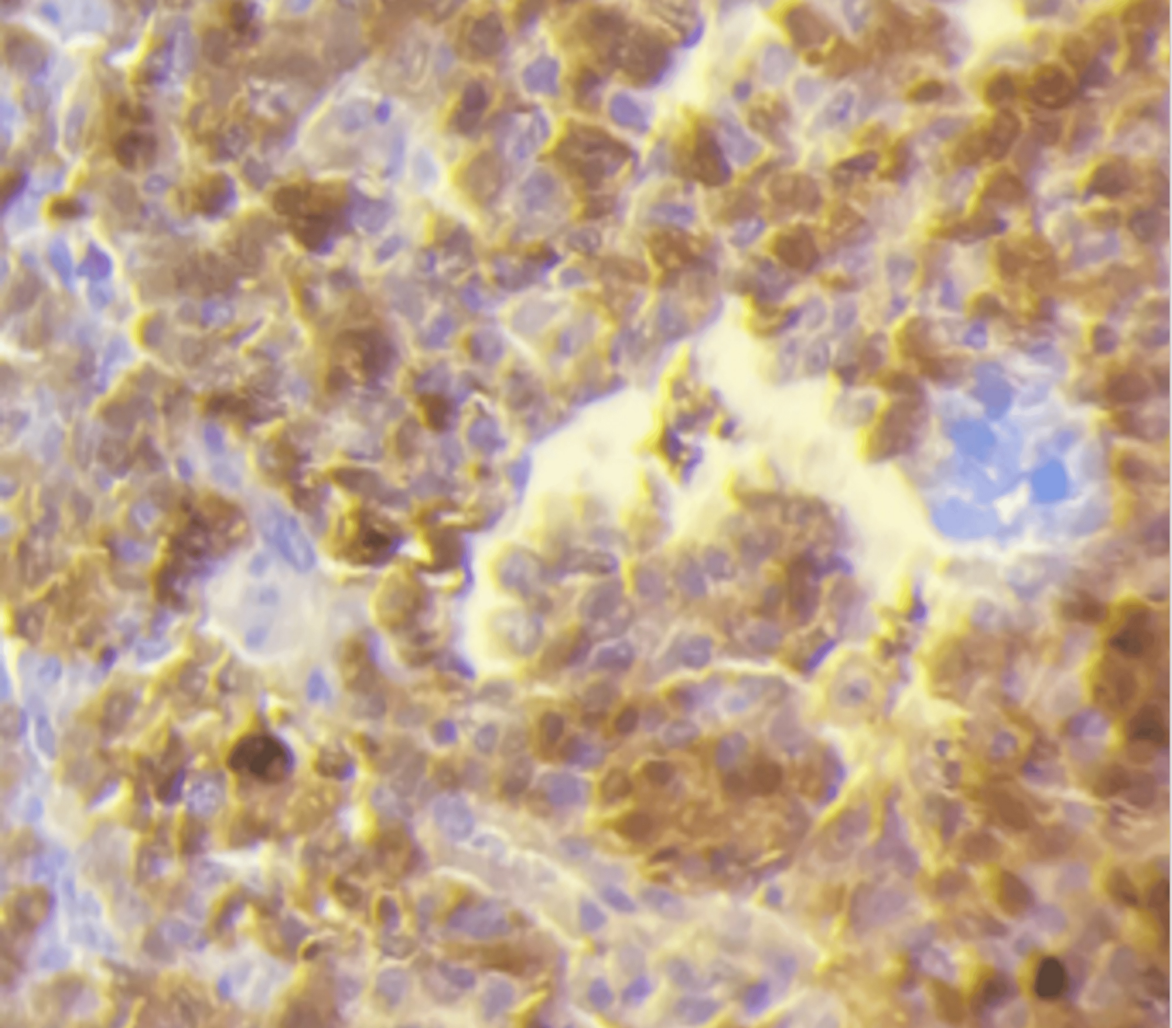
Immunohistochemical stain The image is showing diffuse nuclear positivity for SALL4 (Sal-like protein 4) in yolk sac tumor (40X magnification). SALL4 is a highly sensitive and specific immunohistochemical stain for yolk sac tumor, showing a diffusely finely granular nuclear staining pattern.

**Figure 7:**
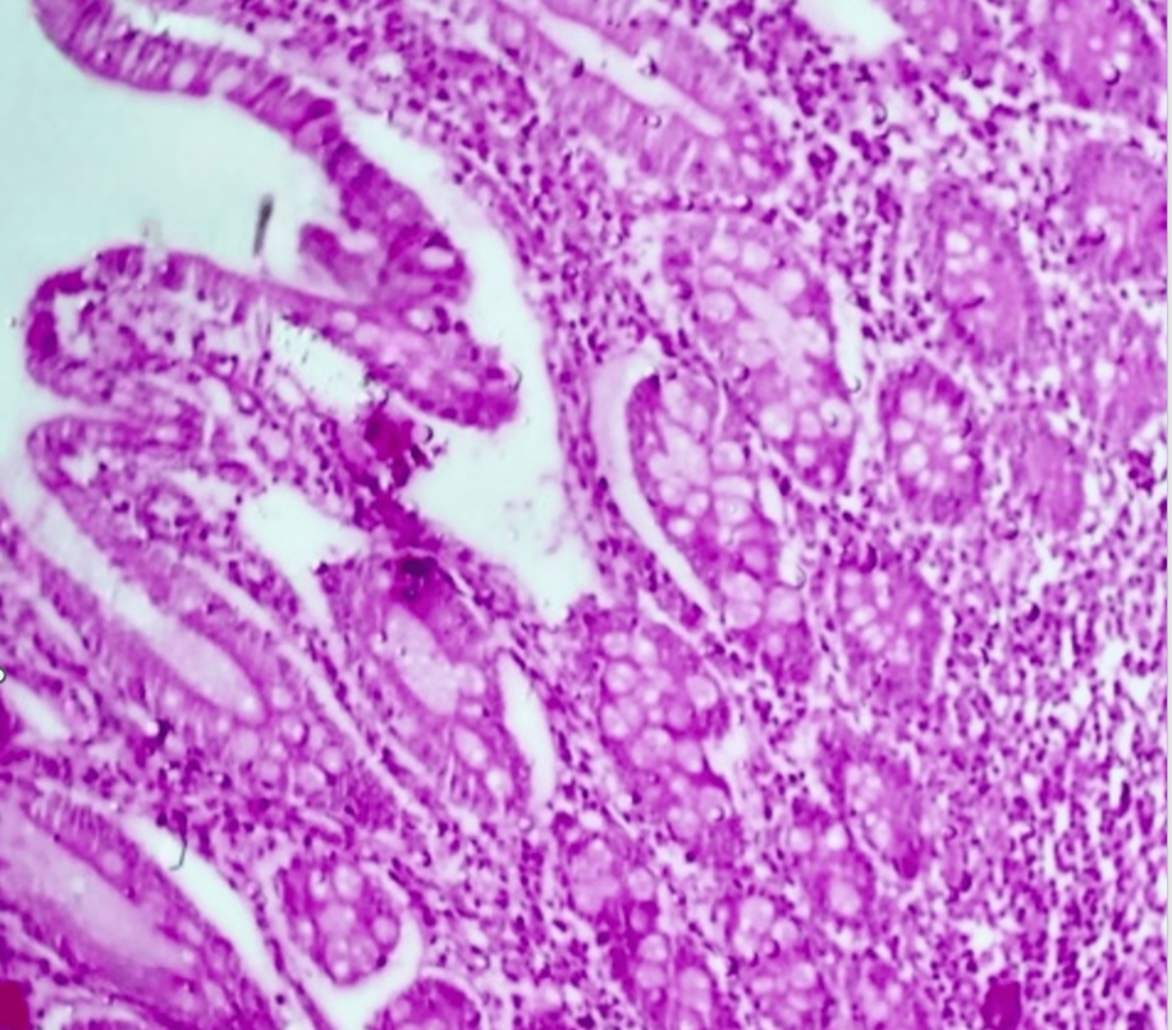
Teratoma showing small intestinal mucosa lined by mucin producing tall columnar epithelium (40X magnification)

**Figure 8:**
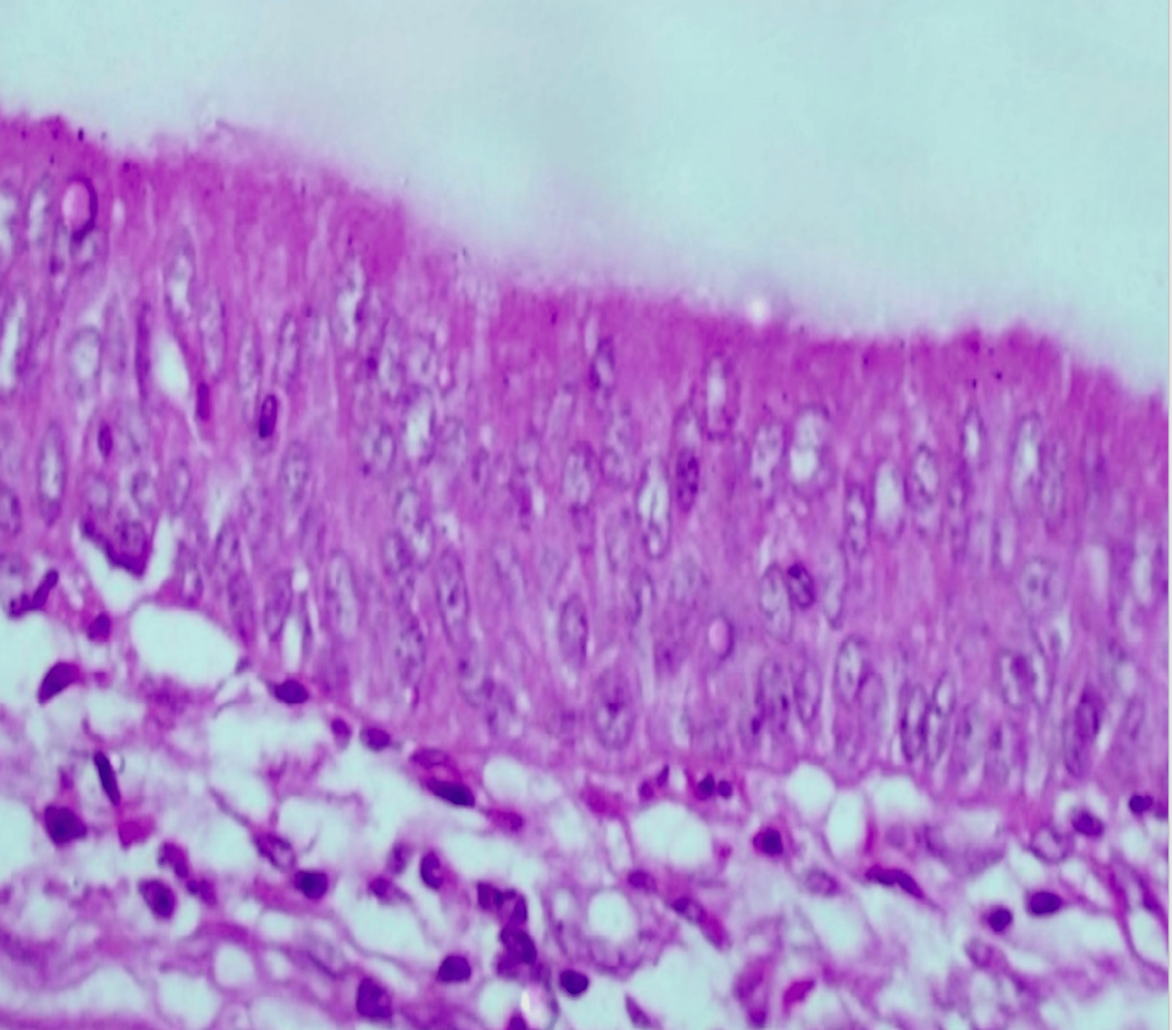
Teratoma showing respiratory mucosa lined by pseudostratified ciliated columnar epithelium (40X magnification)

Histopathological examination revealed cyst walls lined by keratinized stratified squamous epithelium. A portion of the solid area showed proliferation of atypical cells in microcystic, macrocystic, glandular, papillary, and solid patterns. Characteristic Schiller-Duval bodies (papillae with central blood vessels) were seen in some areas. Individual tumor cells exhibited enlarged round to ovoid nuclei with irregular nuclear membranes, vesicular chromatin, prominent nucleoli, and a moderate to abundant eosinophilic to clear cytoplasm. Atypical mitotic figures (12/10 hpf) were present. Focal areas of necrosis were also noted. The remaining areas revealed normal tissues derived from all three germ layers: ectoderm, endoderm, and mesoderm, comprising skin, respiratory epithelium, gastric tissue, pancreatic tissue, and adipose tissue. Immunohistochemistry demonstrated positivity of the malignant yolk sac component for SALL4 (Sal-like protein 4), Glypican-3, and AFP, which confirmed yolk sac tumor differentiation. The tumor was negative for octamer-binding transcription factor 3/4 (OCT3/4) and cluster of differentiation 30 (CD30), findings that distinguished it from embryonal carcinoma. This immunoprofile, characterized by SALL4 and Glypican-3 positivity with absence of OCT3/4 and CD30 expression, was highly specific for yolk sac tumor. Thus, a diagnosis of mature teratoma with yolk sac tumor component was made. Histopathological findings are shown in Figures 4-8. 

Postoperatively, the patient was kept on regular oncologic follow-up. Serum AFP was monitored every month for the first six months, then every three months for the next 18 months, along with periodic abdominal imaging as indicated. Adjuvant chemotherapy with the JEB regimen (carboplatin, etoposide, and bleomycin) was administered for three cycles following complete surgical excision. Clinical evaluations were conducted every two to three months during the first year and at longer intervals thereafter. Over two years of follow-up, the child remained clinically well with no evidence of recurrence, and the most recent AFP was 2.54 mg/dL, within the normal range. Long-term surveillance continues to monitor for late treatment-related toxicities and to ensure normal growth and development. 

## Discussion

Teratomas can be completely benign, such as mature teratomas, or potentially malignant if they contain immature elements. Some teratomas may also harbor frankly malignant components. Mature teratomas typically contain well-differentiated tissues derived from all three germ layers, such as bone, teeth, cartilage, fat, nerve tissue, skin and its appendages, along with gastrointestinal and respiratory epithelium. An immature teratoma, on the other hand, may contain both adult and embryonic tissue, including neuroectoderm and neuroepithelium. Malignant transformation within teratomas is rare but can occur in pre-existing teratomatous elements, for example, rhabdomyosarcoma (most common), adenocarcinoma, or primitive neuroectodermal tumor. It may also result from differentiation of totipotential germ cells with concomitant malignant transformation, as seen in the present case, termed a mixed germ cell tumor [5]. Teratomas that undergo malignant transformation comprise a small proportion of all teratomas [6].

Extragonadal intraperitoneal teratomas are quite rare. They have been reported in the stomach, liver, mesentery, omentum, and other intraperitoneal tissues, or as isolated lesions. To date, fewer than 50 cases of liver teratomas, fewer than 25 cases of mesenteric teratomas (primarily documented as single case reports), 47 cases of omental teratomas, and 102 cases of gastric teratomas have been reported [7-10]. In the present case, the tumor did not arise from any of these structures and appeared instead as a unique, isolated intraperitoneal mass in the left upper quadrant of the abdomen. Such primary isolated intraperitoneal teratomas, not originating from any visceral tissue, are exceptionally rare, with only one previously reported case [11]. The current case is further unique due to the presence of a yolk sac tumor component in an extragonadal teratoma, whereas most reports describe yolk sac components arising in gonadal teratomas [12]. Importantly, the presence of a malignant component within a benign mature cystic teratoma drastically alters both prognosis and management. This underscores the importance of meticulous HPE, as small malignant foci can be missed. Therefore, large mass lesions should be generously sampled to ensure accurate diagnosis.

The diagnosis of a yolk sac tumor component within a teratoma is based on identifying characteristic microscopic patterns, such as microcystic, reticular, or endodermal sinus-like areas, supported by immunohistochemistry. Yolk sac component typically exhibits strong cytoplasmic and membranous positivity for Glypican-3 and AFP, both sensitive markers of yolk sac differentiation. The highly sensitive and specific marker SALL4 shows diffuse nuclear positivity, confirming germ cell origin, while cytokeratin (AE1/AE3) is commonly positive. In contrast, OCT3/4 and CD30 are negative, which helps distinguish it from embryonal carcinoma. The combined histomorphologic and immunohistochemical findings confirm the diagnosis of a yolk sac tumor component within a teratoma [13].

The prognosis of pediatric germ cell tumors generally worsens with increasing age at onset. Teratomas in older children are more likely to harbor yolk sac tumor components, and tumors with immature elements at the time of resection carry a higher risk of recurrence. Conversely, prognosis in younger children is relatively favorable, with minimal recurrence after complete resection [14]. In the present case of a mature cystic teratoma with a yolk sac component, the child had an abdominal mass since the age of two and presented to us at age three. While young age and the mature teratoma element impart a good prognosis, the yolk sac component poses a risk for metastasis and recurrence. Fortunately, complete surgical excision was possible, as the tumor was localized and non-infiltrating. Postoperatively, the child received three cycles of JEB chemotherapy (etoposide, carboplatin, and bleomycin). Although cisplatin-based regimens have traditionally been the standard for malignant germ cell tumors, the JEB regimen is increasingly favored to avoid the neurotoxicity and ototoxicity commonly associated with cisplatin. The child remained disease-free with no evidence of tumor recurrence or metastasis during two years of follow-up.

Malignant transformation can occur in 2-6% of cases of GCT with teratomatous differentiation [9,15]. Malignant transformation arising from the somatic component of teratoma is often resistant to standard chemotherapy but may respond to tailored regimens based on the specific histologic type [15]. Thus, surgical resection remains the cornerstone of treatment in such cases. For patients with metastases or unresectable tumors, management typically involves maximal permissible resection combined with adjuvant chemotherapy, or chemotherapy alone, guided by the histology of the malignant component. In contrast, when malignant elements arise from totipotential germ cells, as seen in the present case, the tumor generally responds well to cisplatin-based or JEB-based chemotherapy. Therefore, the standard approach involves surgical excision followed by chemotherapy, with or without radiotherapy [16]. Ultimately, thorough sampling of large tumors and careful HPE to detect malignant components are critical for guiding appropriate management of teratomas.

## Conclusions

This case highlights the essential role of histopathological evaluation by a pathologist in identifying a rare primary isolated intraperitoneal teratoma with a malignant yolk sac component. Because these tumors are extremely uncommon, thorough gross and microscopic examination is crucial, as small malignant areas can be concealed within an apparently benign mass. Accurate diagnosis relies on adequate tissue sampling, detailed histopathologic review, and immunohistochemical confirmation of germ cell differentiation. Detection of a yolk sac component significantly changes the patient’s management, requiring complete surgical excision followed by postoperative chemotherapy. Regular follow-up is vital for early identification of recurrence. Monitoring should include monthly AFP levels during the first six months, which is the period of highest recurrence risk, followed by testing every three months for the next 18 months. Clinical examinations are advised every two to three months during the first year and at longer intervals thereafter, with imaging as indicated. Early diagnosis and vigilant follow-up ensure excellent outcomes.
